# Structures of PPARγ complexed with lobeglitazone and pioglitazone reveal key determinants for the recognition of antidiabetic drugs

**DOI:** 10.1038/s41598-017-17082-x

**Published:** 2017-12-04

**Authors:** Min A Lee, Lingchen Tan, Huiseon Yang, Yeong-Gwan Im, Young Jun Im

**Affiliations:** 1 College of Pharmacy, Chonnam National University, Gwangju, 61186 Republic of Korea; 2 Department of Oral Medicine, Chonnam National University Dental Hospital, Gwangju, 61186 Republic of Korea

## Abstract

Peroxisome proliferator-activator receptor (PPAR) γ is a nuclear hormone receptor that regulates glucose homeostasis, lipid metabolism, and adipocyte function. PPARγ is a target for thiazolidinedione (TZD) class of drugs which are widely used for the treatment of type 2 diabetes. Recently, lobeglitazone was developed as a highly effective TZD with reduced side effects by Chong Kun Dang Pharmaceuticals. To identify the structural determinants for the high potency of lobeglitazone as a PPARγ agonist, we determined the crystal structures of the PPARγ ligand binding domain (LBD) in complex with lobeglitazone and pioglitazone at 1.7 and 1.8 Å resolutions, respectively. Comparison of ligand-bound PPARγ structures revealed that the binding modes of TZDs are well conserved. The TZD head group forms hydrogen bonds with the polar residues in the AF-2 pocket and helix 12, stabilizing the active conformation of the LBD. The unique *p*-methoxyphenoxy group of lobeglitazone makes additional hydrophobic contacts with the Ω-pocket. Docking analysis using the structures of TZD-bound PPARγ suggested that lobeglitazone displays 12 times higher affinity to PPARγ compared to rosiglitazone and pioglitazone. This structural difference correlates with the enhanced affinity and the low effective dose of lobeglitazone compared to the other TZDs.

## Introduction

Type 2 diabetes mellitus is a progressive metabolic disorder, characterized by hyperglycemia and insulin resistance in peripheral tissue. Insulin resistance is a condition in which cells fail to respond to insulin properly, causing the impaired uptake and utilization of glucose in adipose tissue and skeletal muscle^1^. Type 2 diabetes can be treated by several types of medications that increase insulin secretion by the pancreas, increase the sensitivity of target organs to insulin, reduce excessive hepatic glucose production, increase glucose utilization in the peripheral tissues, and reduce the carbohydrate absorption in the intestines^2^.

Peroxisome proliferator-activated receptors (PPARs) include three subtypes: PPARα, PPARδ, and PPARγ; The PPARs are ligand-activated transcription factors that belong to the superfamily of the nuclear hormone receptors^3^. PPARs heterodimerize with the retinoid X receptor (RXR) and bind to peroxisome proliferator-response elements (PPREs), altering the transcription of target genes^4^. PPARγ which is mainly expressed in adipose tissue, liver, and skeletal muscle, regulates the genes involved in adipocyte differentiation and lipid metabolism. The endogenous ligands for PPARγ are polyunsaturated fatty acids, oxidized fatty acids, and prostaglandins^4^. Upon agonist binding, the conformation of the ligand binding domain (LBD) is altered to expose the binding cleft for the recruitment of transcriptional coactivators, which initiate the transcription of target genes^5^. Activation of PPARγ increases glucose uptake and utilization in the peripheral organs, stimulates fatty acid storage in adipocytes, enhances insulin signaling, and decreases gluconeogenesis in the liver, thereby improving insulin sensitivity^6^.

Thiazolidinediones (TZDs) including pioglitazone and rosiglitazone are synthetic antihyperglycemic agents that act as PPARγ agonists^6^. TZDs enhance insulin action and improve hyperglycemia in patients with type 2 diabetes^3^. All TZDs have similar effects on glycemic control, and a range of adverse effects, such as weight gain, fluid retention, and increased risk of heart failure, which seem to be PPARγ-mediated^7^. Some adverse effects of TZDs are considered clinically significant. Rosiglitazone is no longer widely used owing to increased risks of myocardial infarction and cardiovascular mortality, though the FDA removed the restrictions on rosiglitazone after reviewing clinical data^8,9^. Use of pioglitazone has been suspended in some European countries due to its possible association with bladder cancer^10^.

**Figure 1:**
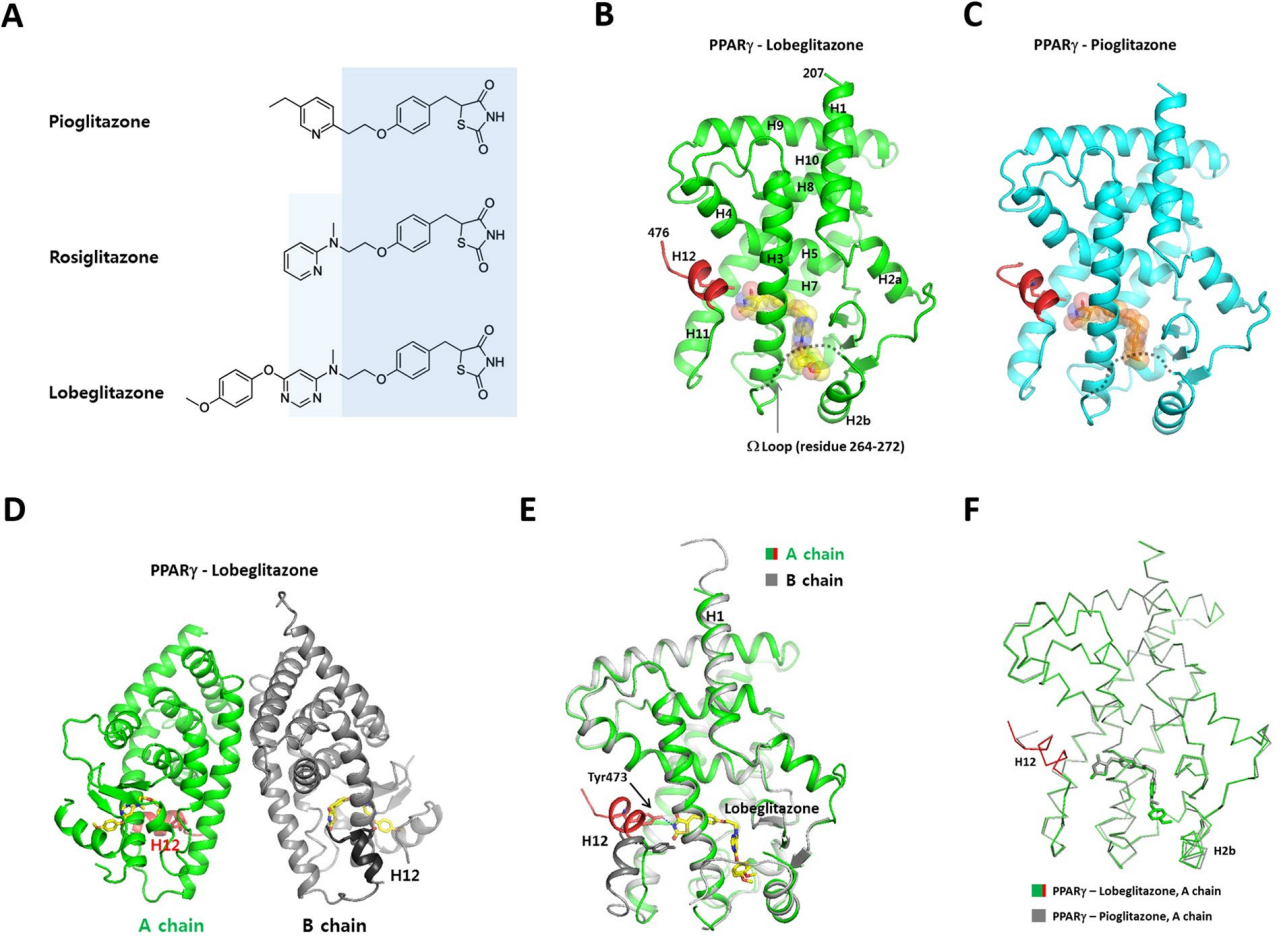
Overall structures of the PPARγ LBD complexed with pioglitazone and lobeglitazone. (**A**) Chemical structures of pioglitazone, rosiglitazone, and lobeglitazone. Structurally conserved parts of TZDs are shaded with light blue colors. (**B**) Monomeric structure of the PPARγ LBDs complexed with lobeglitazone. The C-terminal AF-2 helix (H12) is colored in red. The disordered Ω-loops are indicated with dashed lines. The bound ligands are shown as transparent spheres. (**C**) Monomeric structure of the PPARγ LBDs complexed with pioglitazone. (**D**) The PPARγ LBD crystallized as a homo-dimer composed of active and inactive forms in an asymmetric unit. (**E**) Structural superposition of A and B chains of the lobeglitazone-bound PPARγ LBD. (**F**) Structural comparison of the lobeglitazone- and pioglitazone-bound PPARγ LBDs.

Lobeglitazone (trade name Duvie; Chong Kun Dang Pharmaceutical Corporation) was developed as a more effective and safe antidiabetic TZD drug. Lobeglitazone was conceptually designed by modification of the rosiglitazone structure with a substituted pyrimidine. Lobeglitazone has a *p*-methoxyphenoxy group at the 4-position of the pyrimidine moiety^11^ (Fig. 1A). Lobeglitazone showed more potent activity than the reference compounds (pioglitazone and rosiglitazone) with an EC_50_ value of 0.018 μM in a type 2 diabetes animal model, which is 16 times lower than pioglitazone (EC_50_ 0.30 μM)^11,12^. Lobeglitazone exhibited similar efficacy profiles in glycemic control and lipid modulation to pioglitazone, but with a 30 times smaller dose in clinical studies^13,14^. The molar amount of lobeglitazone (Duvie^R^, 0.415 mg/tab.) contained in a single tablet is 14 times less than that of rosiglitazone (Avandia^R^, 6.04 mg/tab.) and 32 times less than that of pioglitazone (Actos^R^, 13.6 mg/tab.), indicating the high potency of lobeglitazone. In addition, lobeglitazone displayed significantly reduced side effects regarding cardiovascular disease and bladder cancer^15,16^. Currently, three TZDs (rosiglitazone, pioglitazone, and lobeglitazone) are available for the treatment of type 2 diabetes. The structures of rosiglitazone-bound PPARγ were previous determined revealing a binding mode of the TZD drug^5,17–19^. Despite the effectiveness of TZD drugs for treating insulin resistance, the crystal structures of pioglitazone and lobeglitazone-bound PPARγ have not been reported. How the small modification introduced in lobeglitazone leads to the dramatic increase in potency and reduction of side effects is not known clearly.

In this study, to delineate the binding modes of TZDs to PPARγ and to identify the structural determinants for the high potency of lobeglitazone compared to other TZD drugs, we determined the high resolution crystal structures of the PPARγ LBD in complex with lobeglitazone and pioglitazone. Comparison of ligand-bound PPARγ structures revealed a conserved binding mode of TZDs to PPARγ, confirming the same therapeutic effects of TZD drugs. However, the unique *p*-methoxyphenoxy group of lobeglitazone makes additional hydrophobic contacts with the ligand-binding pocket, which correlates with the enhanced affinity and the low effective dose of lobeglitazone compared to those of rosiglitazone and pioglitazone. Consistently, lobeglitazone stabilizes the PPARγ LBD against thermal denaturation substantially more than other TZDs, indicating a high affinity binding of lobeglitazone. Computational docking analysis using the structures of TZD-bound PPARγ suggests that lobeglitazone displays 12 times higher affinity to PPARγ than rosiglitazone and pioglitazone. This study expands the understanding on the structural basis of PPARγ-TZD drug interactions.

## Results

### Overall structure of PPARγ complexed with lobeglitazone and pioglitazone

To understand the structural basis of PPARγ - TZD interaction, we crystallized the lobeglitazone- and pioglitazone-bound PPARγ ligand-binding domains (PPARγ LBDs) and determined the structures of the complexes at 1.7 and 1.8 Å resolutions, respectively. The structures were determined by molecular replacement using the structure of the rosiglitazone-bound PPARγ LBD^19^ (PDB code: 4EMA) and refined the structures with the R_free_ values of 22.3 and 23.2%, respectively. The crystallographic statistics are shown in Table 1. The structures of the PPARγ-ligand complex belong to the monoclinic space group P2_1_, and contain a dimer in the asymmetric unit. The PPARγ LBD was a monomer in solution when analyzed by size exclusion chromatography during protein purification, suggesting that the homodimer in the crystal was formed during crystallization process. Most residues in the PPARγ LBDs were well defined in the electron density maps, except for the mobile loops between H2b and H3. The final model of PPARγ-lobeglitazone consists of two monomers of the PPARγ LBD (referred as A and B chains), two molecules of lobeglitazone, and 454 water molecules. The PPARγ LBD is composed of 13 helices arranged into a three-layered sandwich and a three-stranded β-sheet, with the C-terminal AF-2 helix (H12) (Fig. 1B,C). The overall fold of the TZD-bound PPARγ LBD shows good agreement with the reported structures of PPARγ LBDs. The A-chain corresponds to the canonical active conformation of the PPARγ LBD with H12 in an active position, and the B-chain has an inactive conformation with H12 protruding away from the molecule (Fig. 1D). The inactive conformation of H12 in chain B seems to be caused by the protein packing interaction in the crystal lattice^20^. The conformations of chain A and chain B are almost identical, except for the C-terminal H12 (Fig. 1E).

**Table 1:** Data-collection and refinement statistics.

Crystal	PPARγ-lobeglitazone complex	PPARγ-pioglitazone complex
Construct	Residues 207–477	Residues 207–477
Data collection		
Beamline	PLS-7A	PLS-5C
Wavelength (Å)	0.97950	0.97950
Space group	*P*2_1_	*P*2_1_
Unit-cell parameters (Å, °)	*a* = 56.3 Å, *b* = 88.5 Å, *c* = 58.0 Å, β = 89.8°	*a* = 55.6 Å, *b* = 88.1 Å, *c* = 57.7 Å, β = 91.2°
Resolution limit (Å)	50–1.7 (1.735–1.70)	50–1.8 (1.83–1.80)
No. of reflections	252147	222377
No. of unique reflections	62190 (3058)	49957 (2257)
Multiplicity	4.1 (4.1)	4.5 (4.2)
Mean *I*/*σ*(*I*)	33.3 (4.6)	33.4 (3.5)
Completeness (%)	99.8 (99.8)	97.1 (97.2)
*R*_merge_ (%)	7.9 (37.6)	6.5 (38.2)
*R*_p.i.m._ (%)	3.8 (17.8)	3.1 (18.5)
Wilson *B* factor (Å)	23.4	30.1
Refinement		
*R*_work_ (%)	19.2 (22.4)	20.2 (24.5)
*R*_free_ (%)	22.3 (29.9)	23.2 (31.2)
R.m.s.d., bond lengths (Å)	0.007	0.007
R.m.s.d., bond angles (°)	0.932	0.839
*B* factor (Å^2^)		
Overall	30.32	40.8
protein chain A (chain B)	25.6 (30.8)	40.8 (40.3)
ligand molecule A (molecule B)	29.6 (46.4)	47.9
Water	36.7	43.8
No. of non-H atoms		
Protein (ligand)	4183 (64)	4183 (25)
Solvent	677	453
Ramachandran statistics		
Favored (%)	99.0	98.6
Disallowed (%)	0.19	0.20
PDB entry	5Y2T	5Y2O

The loop connecting H2b and H3, which is referred to as the Ω-loop, was poorly defined in the electron density maps in both lobeglitazone and pioglitazone-bound PPARγ LBDs. The Ω-loop is thought to be a very flexible region of LBD, serving as a gate to the ligand binding pocket^21^. The Ω-loop is more ordered in chain B, which results from stabilization of the loop by lattice contacts in the crystal.

**Figure 2:**
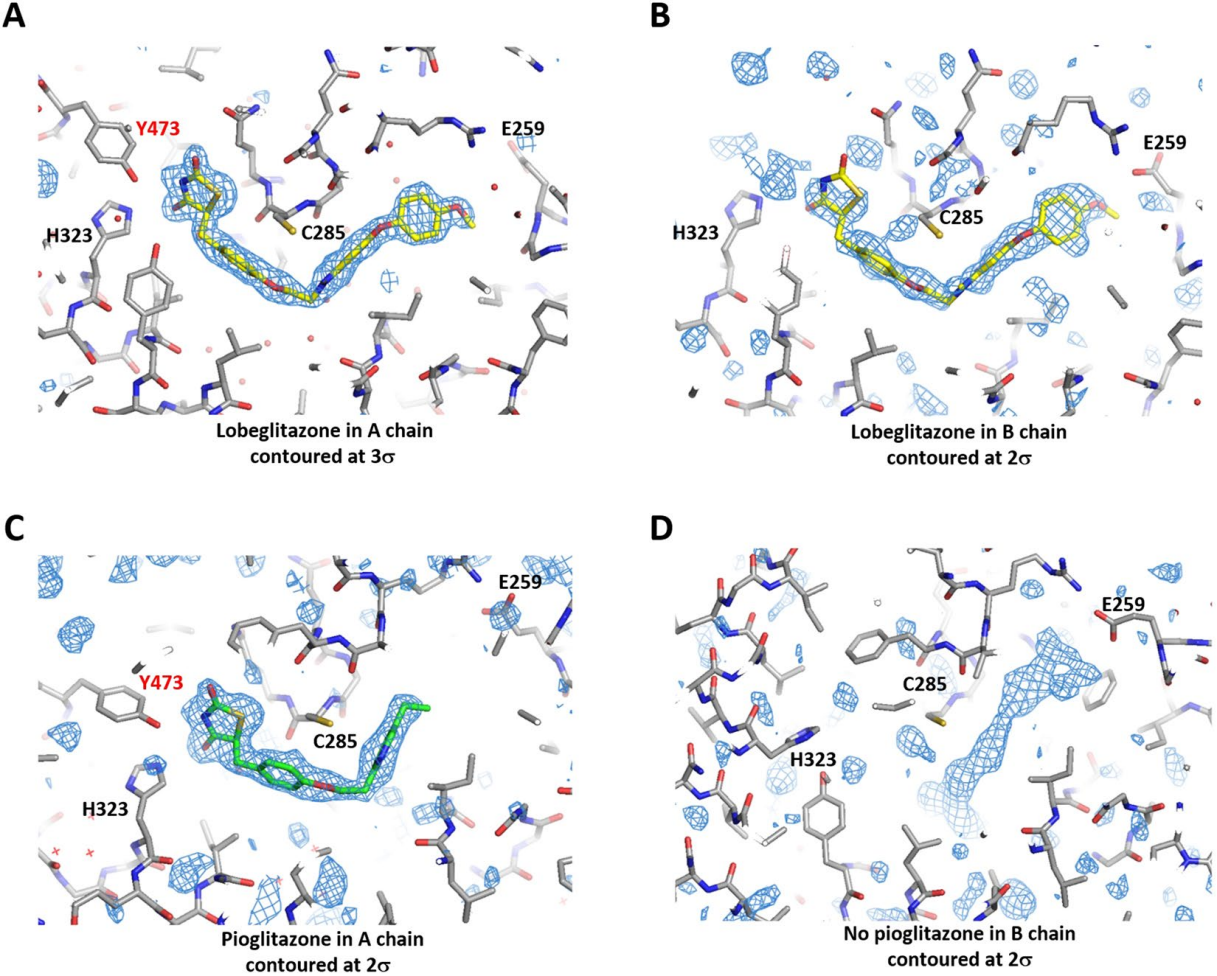
Electron density maps of lobeglitazone and pioglitazone in the ligand-binding pockets of PPARγ. (**A**) 1.7 Å resolution *F*_o_-*F*_c_ maps of lobeglitazone in A chain with the final model superimposed. (**B**) 1.7 Å resolution *F*_o_-*F*_c_ maps of lobeglitazone in B chain. (**C**) 1.8 Å resolution *F*_o_-*F*_c_ maps of pioglitazone in A chain. (**D**) 1.8 Å resolution *F*_o_-*F*_c_ maps of the ligand-binding site in B chain of the pioglitazone-bound PPARγ LBD.

The structure of the PPARγ - pioglitazone complex has an identical space group with very close cell parameters to the lobeglitazone-bound structure. The structures of the PPARγ LBD complexed with lobeglitazone and pioglitazone are very similar to each other with a root-mean-square deviation (RMSD) of 0.47 Å for the 260 C_α_ atoms of the A chains (Fig. 1F). However, unlike the lobeglitazone molecules presenting in both the A and B chains, pioglitazone was bound to only the A chain of the PPARγ LBD dimer (Fig. 2A–C). Pioglitazone was absent in chain B, which has an inactive conformation of H12. Instead, electron densities resembling those of free fatty acids or polyethylene glycol were weakly visible in the binding pocket of chain B (Fig. 2D). We hypothesized that bacterial fatty acids were incorporated into the hydrophobic pocket of the PPARγ LBD during protein expression and purification. The presence of fatty acids in PPAR LBDs was previously reported in recombinant PPAR protein preparation^22^. It seems that the low affinity of pioglitazone compared to lobeglitazone might not be enough to replace the fatty acids in the B molecule displaying an inactive conformation of H12. The crystal structure of the PPARγ - rosiglitazone complex, which had an identical space group (PDB id: 4EMA), also lacked a rosiglitazone molecule in the B chain^19^.

### Structural basis of lobeglitazone and pioglitazone recognition

**Figure 3:**
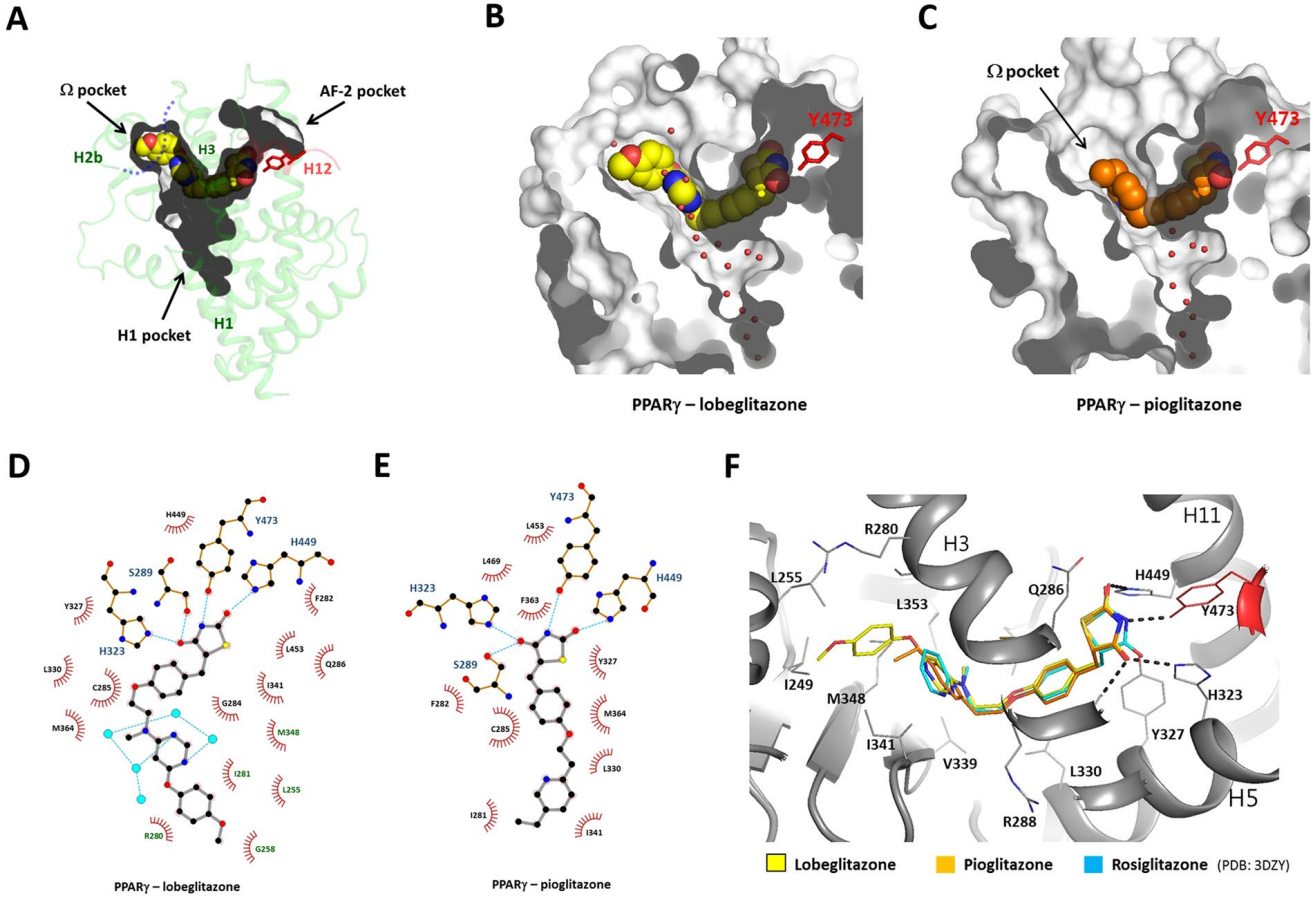
Structure of PPARγ – TZD interaction. (**A**) Surface representation of the Y-shaped ligand-binding cavity of PPARγ. The overall structure of the PPARγ LBD is shown in transparent ribbons. The bound lobeglitazone is shown in yellow spheres. (**B**) The surface representation of the ligand-binding pocket with lobeglitazone. Water molecules in the cavity are shown in small red spheres. (**C**) The surface representation of the ligand-binding pocket with pioglitazone. (**D**) The interaction of lobeglitazone and the PPARγ LBD was shown in 2-dimension using software Ligplot^41^. The residues colored in green indicate the residues in the Ω-pocket. Water molecules are shown in cyan spheres. The hydrogen bonds are shown in dotted cyan lines. (**E**) The interaction of pioglitazone and the PPARγ LBD. (**F**) Structural comparison of the ligand binding modes for lobeglitazone, pioglitazone, and rosiglitazone. The structure of rosiglitazone was taken from the PDB id, 3DZY. The Tyr473 from H12 are colored in red.

The PPARγ LBD has a large Y-shaped ligand binding pocket with a volume of roughly 1,300 Å^3^ (Fig. 3A). The ligand-binding pocket can be divided into three sub-pockets. Hereafter, the sub-pockets near helix 12, the Ω-loop, and helix 1 are referred to as the AF-2, Ω and H1 pockets^23^, respectively. The central region of the ligand-binding pocket of PPARγ is surrounded by mainly nonpolar residues such as Leu330, Leu339, Leu353, and Met364. However, there are clusters of polar residues at both ends of the ligand-binding cavity. The AF-2 pocket is composed of many polar residues such as Cys285, Ser289, His323, Tyr327, His449, and Tyr473. The Ω-pocket contains a few polar residues including Glu259, Arg280, and Ser342. The Ω-pocket is mainly composed of hydrophobic residues from the β-sheet (Ile249, Met348, and Ile341), H2b (Leu255, Gly258, and Ile262), and H3 (Ile281).

The electron densities of lobeglitazone and pioglitazone were clearly visible in the ligand-binding pockets of chain A (Fig. 2A,C). The strong electron densities and the low B factors of lobeglitazone and pioglitazone indicate a high occupancy in the ligand-binding pocket (Table 1). The occupancy refinement using software Phenix showed that the lobeglitazone molecules in A and B chains, and the pioglitazone molecule in A chain, have 96%, 74%, and 82% occupancies, respectively. Lobeglitazone and pioglitazone are completely buried inside the hydrophobic pocket (Fig. 3B,C). Lobeglitazone occupies two sub-pockets (the AF-2 and Ω-pockets) of the Y-shaped ligand-binding pocket. Lobeglitazone, with its molecular volume of 577 Å^3^, occupies 44% of the ligand-binding cavity, which is 10% more than the space occupied by pioglitazone (34%). The contact surface area of lobeglitazone to the ligand-binding pocket is 418 Å^2^, which is 1.33 times larger than the contact area of pioglitazone (314 Å^2^). The lobeglitazone bound to the B chain displays slightly weak electron densities and higher B-factors than the ligand in chain A, owing to the lack of hydrogen bonding to H12. Lobeglitazone and pioglitazone are bound to position their thiazolidinedione groups adjacent to H12, and adopt a U-shaped conformation with the hydrophobic chains wrapping around H3. The thiazolidinedione group makes multiple hydrogen bonds with the polar residues in the pocket. The nitrogen atom of the TZD head group makes a hydrogen bond with the hydroxyl group of Tyr473 in H12, stabilizing the active conformation of H12 (Fig. 3D,E). Two carbonyl groups of the TZD head group make hydrogen bonds with the side chains of His323, Ser289, and His449. The protein-ligand interactions observed in chains A and B were identical, except in the region around H12.

The apo form of the PPARγ LBD has a large internal cavity which can accommodate many water molecules. The tight TZD binding excludes water molecules in the AF-2 pocket and the central region of the hydrophobic cavity. Water molecules are present in the exposed region of the cavity and in the H1-pocket which is unoccupied by the ligand (Fig. 3B). The two nitrogen atoms of pyrimidine ring of lobeglitazone make hydrogen bonds with the two water molecules nearby (Fig. 3D). However, there is no direct water-mediated hydrogen bonds between the ligand and the protein. In the pioglitazone-bound form, there is no water molecules hydrogen bonding to the ligand. The electron densities of water molecules were not visible within the hydrogen-bonding distance to pioglitazone, suggesting that water molecules do not contribute to the specific interaction of TZD binding to the PPARγ LBD.

The binding mode of lobeglitazone is almost identical to rosiglitazone with good superposition of ligand structures in the binding pocket^5^ (Fig. 3F). However, lobeglitazone makes more extensive van der Waals contacts with the ligand-binding pocket than rosiglitazone and pioglitazone. The unique *p*-methoxyphenoxy group of lobeglitazone is positioned in the Ω pocket, making tight hydrophobic interactions with the residues of the pocket walls (Fig. 3D,E). The contacting surface area of the *p*-methoxyphenoxy group is 90 Å^2^, contributing 22% of the contact area of lobeglitazone to the binding pocket. However, there is no direct interaction with the disordered Ω-loop. The residues in the Ω-pocket (Ile249, Leu255, Ile281, Ile341, and Met348) interacting with the *p*-methoxylphenoxy group are relatively rigid, and undergo no conformational changes upon ligand binding. The binding of lobeglitazone to chain B, which displays an inactive conformation of H12, suggests that the extensive hydrophobic interactions significantly contribute to the high affinity of lobeglitazone to PPARγ.

The major difference in pioglitazone compared to rosiglitazone and lobeglitazone is the shorter length of the linker between pyridine and phenyl groups owing to the lack of methylamino group (Fig. 1A). However, the ethyl pyridine group of pioglitazone occupies a similar position to the pyrimidine group of lobeglitazone in the binding pocket (Fig. 3F). The pioglitazone-bound PPARγ LBD displays almost an identical LBD conformation to the lobeglitazone-bound form. This observation suggests that TZD drugs have a conserved binding mode to PPARγ and share the same therapeutic effects via PPARγ activation.

### Lobeglitazone binding stabilizes the PPARγ LBD

We recognized that the high potency of lobeglitazone compared to other TZD drugs could be clearly demonstrated by direct determination of ligand binding affinities to the PPARγ LBD. The binding affinities of lobeglitazone and pioglitazone to the isolated PPARγ LBD were not known. Therefore, we attempted affinity measurements of lobeglitazone and pioglitazone by isothermal titration calorimetry (ITC). The titration of ligand to receptor using ITC requires solubilization of the ligand into a free form. However, lobeglitazone and pioglitazone were almost completely insoluble in the aqueous buffer, which interfered with fast titration of the ligands to the PPARγ LBD. The ligand binding to PPARγ seems a slow process requiring extraction of ligands from the lipid aggregate and a conformational change of the ligand-binding domain. Therefore, as an alternative approach, we estimated the relative affinities of TZDs using thermal shift assays of the PPARγ LBD. It is known that ligand binding by nuclear receptors results in the stabilization of the LBD against thermal denaturation, and that the degree of stabilization correlates with the affinity of the ligand^24^.

**Figure 4:**
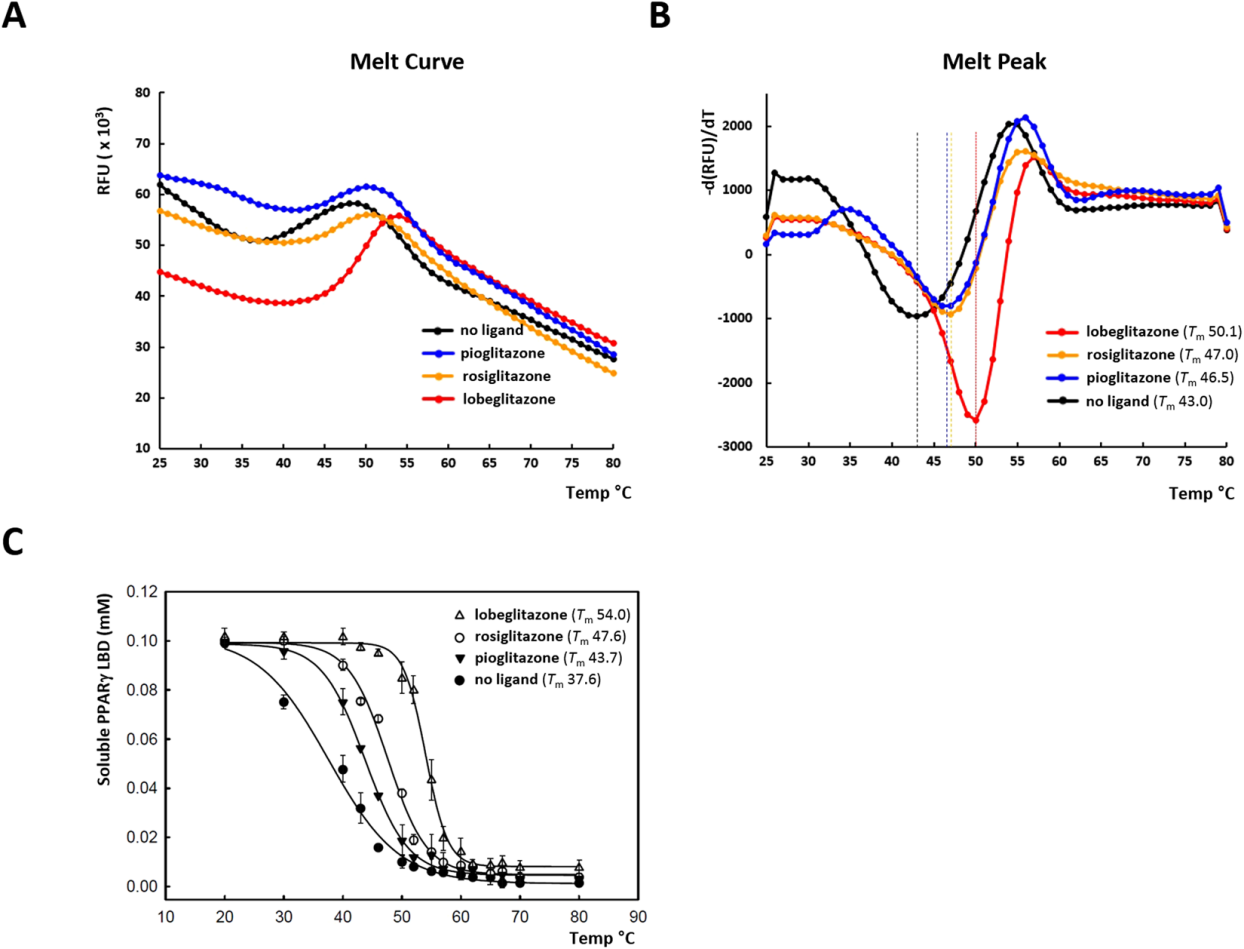
Stabilization of the PPARγ LBD by ligand binding against heat denaturation. (**A**) The melting curves of the PPARγ LBD were monitored by differential scanning fluorimetry with SYPRO orange dye to examine the stabilization by ligand binding from thermal denaturation. (**B**) The reciprocal derivative plots of the melting curves. The dotted lines in the melt peak plots indicate *T*_m_ values. (**C**) Stabilization of the PPARγ LBD against heat denaturation monitored by UV absorbance. The concentration of soluble PPARγ LBD was measured after heat denaturation from the starting protein concentration of 0.1 mM. The data points are means of three independent experiments and the error bars denote standard deviations.

The stabilization of the PPARγ LBD by ligand binding was monitored by increase of melting temperature (*T*_m_) using differential scanning fluorimetry (DFS)^25^. For this purpose, the purified PPARγ LBD in the presence of SYPRO orange dye was heat-treated by gradual increase from 25 to 80 °C using a real-time PCR machine. Melting temperatures were calculated from melting curves by analyzing the fluorescence data (Fig. 4A,B). The *T*_m_ value of apo PPARγ LBD was 43 °C. The pioglitazone and rosiglitazone binding increased the *T*_m_ values to 46.5 and 47.0 °C, respectively. Most of all, lobeglitazone binding showed a substantial increase of the *T*_m_ value to 50.1 °C. This data suggest that lobeglitazone seems to be an excellent stabilizer of the PPARγ LBD compared to the other TZD drugs.

To confirm the results obtained from the DFS experiments, we performed an additional thermal shift assay, which does not require a fluorescent dye. We measured the concentration of soluble protein after heat denaturation of the PPARγ LBD loaded with three different TZDs (Fig. 4C **)**. The apo PPARγ LBD lost solubility by protein precipitation in a highly cooperative manner with a midpoint temperature (*T*_m_) of 38 °C. When bound to the agonist pioglitazone, the *T*_m_ value increased to 44 °C. Rosiglitazone displayed a similar stabilization of the PPARγ LBD to pioglitazone with a *T*_m_ of 48 °C. Notably, the PPARγ LBD complexed with lobeglitazone was significantly stabilized with a *T*_m_ of 54 °C, which is 10 °C higher than the *T*_m_ of the pioglitazone-bound PPARγ LBD. There is a discrepancy of absolute *T*_m_ values between the two thermal shift techniques, which might be caused by the differences of detection methodology and concentration of protein and ligand. However, the patterns of thermal stabilization by the TZDs are consistent with the order of lobeglitazone, rosiglitazone, and pioglitazone, from the highest to lowest *T*_m_ values. Thus, lobeglitazone seems to be an excellent stabilizer of the PPARγ LBD, indicating a higher affinity to PPARγ than the other TZDs tested.

### Affinity estimation of TZDs by computational docking

Computational docking is used to predict bound conformations and free binding energies of small molecule ligands to macromolecular targets^26^. Docking is useful for the study of biomolecular interactions and structure-based drug design^27^. Although the calculation of the absolute binding energy from the structures of a ligand-bound state might be inaccurate, the relative ranking of binding energies is generally reliable for the binding of closely related ligands to identical receptors. The TZD drugs have similar chemical structures and share a conserved binding mode to PPARγ. Since the high-resolution crystal structures of the PPARγ LBD bound with lobeglitazone, pioglitazone, and rosiglitazone are available from this work and previous studies, we excluded the uncertainty caused by the structural flexibilities of ligands and receptors by treating the molecules as rigid bodies during docking analysis. The free binding energies of different TZDs in the PPARγ LBD were calculated using AutoDock Vina^28^ (Table 2). Since the absolute K_d_ value for each compound might not be reliable, we compared the relative K_d_ values using lobeglitazone as a reference. Lobeglitazone displays 12 and 14 times higher affinities than pioglitazone and rosiglitazone, respectively. The test molecule lacking the *p*-methoxyphenoxy group from lobeglitazone has a 9.1 times weaker binding affinity, suggesting the significant contribution of the side group in the strong ligand binding. The results of the docking analysis correlates well with x-ray structures and the thermal denaturation tests, confirming the enhanced affinity of lobeglitazone compared to other TZD drugs.

**Table 2:** Estimation of relative affinities of TZDs to the PPARγ LBD. The relative K_d_ was calculated by AutoDock Vina using the structures of TZD-bound forms.

Molecule	Lobeglitazone	Lobeglitazone excluding *p*-methoxy phenoxy group	Pioglitazone	Rosiglitazone
Affinity (kCal/mol)	−8.40	−7.06	−6.89	−6.79
gauss 1	113.59	94.19	87.07	87.64
gauss 2	1947.3	1473.38	1503.36	1494.2
repulsion	4.569	4.423	5.304	5.329
hydrophobic	26.89	16.75	38.28	26.29
hydrogen	3.637	3.637	3.350	3.916
**Relative affinity (K**_**d**_ **)**	**1**	**9.1**	**12.0**	**14.1**

## Discussion

Type 2 Diabetes mellitus can be treated by several types of medications such as insulin, biguanides, sulfonylureas, and TZDs. During the last decade, a number of new antidiabetic drugs have been introduced such as glucagon-like peptide 1 (GLP-1) analogs, dipeptidyl peptidase-4 (DPP-4) inhibitors, bile acid sequestrants, and sodium glucose transport protein-2 inhibitors^9^. Though the TZDs are effective medications that can treat insulin-resistant diabetes, the use of prototypic TZDs such as rosiglitazone and pioglitazone is declining owing to safety concerns regarding cardiovascular mortality and bladder cancer^9^. Recently, lobeglitazone was developed as a potent TZD with enhanced efficacy and low side effects, and it was approved by the Ministry of Food and Drug Safety (Korea) in 2013.

Adverse effects of drugs are categorized as on-target, chemical-based, or off-target effects^29^. On-target refers to exaggerated pharmacologic effects at the target of interest. Several side effects of TZDs such as weight gain, fluid retention, and increased risk of heart failure seem to be PPARγ mediated. Chemical-based toxicity is related to the physicochemical characteristics of a compound and the metabolites, and their effects on cellular components and/or metabolic pathways. Off-target effects arise from modulation of other unrelated targets. For example, pioglitazone was known to inhibit human monoamine oxidase B (MAO-B) by specifically occupying both substrate-binding and active sites^30^. In addition, TZDs such as pioglitazone and rosiglitazone were suggested to modulate the mitoNEET (novel mitochondrial protein), mTOT (mitochondrial target of thiazolidinones) and glucocorticoid receptor^31–33^. Still, the molecular mechanism of the various side effects of individual TZDs are not well understood. Unlike pioglitazone, lobeglitazone has no risk of bladder cancer, because urinary excretion of lobeglitazone is negligible in humans^12,13^.

In this study, to delineate the binding modes of TZDs to PPARγ and the structural determinants for the high potency of lobeglitazone, we determined the high-resolution structures of the PPARγ LBD in complex with lobeglitazone and pioglitazone. The overall structures of the PPARγ LBD remained identical among the three TZD (rosiglitazone, pioglitazone, and lobeglitazone)-bound receptor complexes, indicating a conserved mode of PPARγ activation. The head groups of the TZDs form a hydrogen bond with Tyr473 of H12, stabilizing the AF-2 of PPARγ in an active conformation, which correlates with full agonism of the drugs^34,35^. The ligand-binding cavity of PPARγ (1,300 Å^3^) is much larger than the cavities of other nuclear receptors, which typically have volumes ranging from 600 Å^3^ to 1,100 Å^3^ ^24^. This feature seems to make the PPARγ LBD intrinsically mobile in solution and allow the presence of many different conformations in its unbound state^36,37^. Therefore, the degree of stabilization of the LBD in its active conformation by ligand binding correlates with the potency of the ligand^36,38^. We observed that lobeglitazone occupied a large volume in the binding cavity and stabilized the PPARγ LBD against thermal denaturation substantially more than other TZDs, indicating high affinity binding of lobeglitazone. The *p*-methoxyphenoxy group of lobeglitazone makes additional hydrophobic contacts with the ligand-binding pocket, which contributes to the enhanced affinity of lobeglitazone. Consistently, computational docking analysis suggested that lobeglitazone displays 12 times higher affinity to PPARγ than rosiglitazone and pioglitazone.

In conclusion, the significant improvement in binding affinity and specificity of lobeglitazone seems to allow a low effective dose, thereby decreasing the adverse effects caused by chemical-based toxicity and off-target effects. This structural study provides a structural basis for PPARγ-TZD interactions and an explanation for the enhanced affinity of lobeglitazone over other TZD drugs.

## Materials and Methods

### Reagents

The full-length PPARγ clone in a pCMV-SPORT6 plasmid (clone ID: hMU000317) was obtained from Korea Human Gene Bank, Medical Genomics Research Center, KRIBB, Korea. Lobeglitazone sulfate (Duvie^R^ tablets) and pioglitazone hydrochloride (Glyact^R^ tablets) were purchased from Chong Kun Dang pharmaceutical co. and Myungmoon pharmaceutical co., respectively. Rosiglitazone and pioglitazone with purity higher than 98% were obtained from Tokyo Chemical Industry Co.

### Cloning and protein purification

DNA encoding the ligand-binding domain (residue 207–477) of human PPARγ (GenBank accession number: NM_138711) was amplified from the full-length clone by polymerase chain reaction (PCR). The primers for the PPARγ LBD were 5′-GTATTA GGATCC GAGTCCGCTGACCTCCG-3′ (forward) and 5′-GATACA CTCGAG CTAGTACAAGTCCTTGTAGATC-3′ (reverse). The PCR products were sub-cloned into the BamHI and XhoI sites of a modified pET28b vector. The PPARγ LBD was tagged with an N-terminal hexahistidine followed by a thrombin protease cleavage site (LVPR/GS). The plasmid containing the PPARγ LBD was transformed to *Escherichia coli* strain BL21(DE3).

*E. coli* cells transformed with the plasmid encoding the PPARγ LBD were grown to an OD_600_ of 0.6 at 310 K in Luria-Bertani medium. Cells were induced by adding isopropyl β-D-1-thiogalactopyranoside to a final concentration of 0.3 mM, and were incubated for 12 hours at 289 K prior to harvesting. The cells expressing the PPARγ LBD were re-suspended in 3X phosphate buffered saline (PBS) containing 30 mM imidazole and lysed by sonication. After centrifugation at 13,000 rpm for 45 min, the supernatant containing the PPARγ LBD was applied to a Ni-NTA affinity column. The protein was eluted from the column using 0.1 M Tris–HCl pH 7.0, 0.3 M NaCl, 0.3 M imidazole. To obtain PPARγ complexed with lobeglitazone and pioglitazone, three tablets of Duvie^R^ and Glyact^R^ were dissolved into the elution buffers containing the PPARγ LBD to final ligand concentrations of 60 μM and 2.9 mM, respectively. The protein was concentrated to 10 mg/ml, and the his-tag was removed by cleavage with thrombin protease with a 2-hour incubation at room temperature. The ligand-bound PPARγ LBDs were subjected to size exclusion chromatography (SEC) using a Superdex 200 column (GE Healthcare) which was pre-equilibrated with 20 mM Tris–HCl pH 7.5, 150 mM NaCl. To sustain the ligand-bound forms of the PPARγ LBDs during SEC, additional Duvie^R^ and Glyact^R^ tablets were dissolved into the equilibration buffers to final concentrations of 5.2 μM lobeglitazone and 229 μM pioglitazone, respectively. The peak fractions containing the ligand-bound PPARγ LBDs were concentrated to 30 mg/ml for crystallization. The PPARγ LBD loaded with rosiglitazone was prepared by the same procedures. Rosiglitazone was added into the affinity and SEC eluted fractions with a final concentration of 60 μM.

### Crystallization and crystallographic analysis

Preliminary crystallization experiments for the PPARγ LBD-ligand complex were carried out at 295 K in 96-well crystallization plates using a multichannel pipette and customized crystallization screening solutions by dispensing 0.8 μl protein solution and 0.8 μl precipitant solution. Initial crystals of the PPARγ LBD appeared after 5 days using a solution consisting of 0.1 M HEPES–NaOH pH 7.0, 20% PEG 8000. The crystallization conditions were further optimized using the hanging-drop technique in 15-well screw-cap plates. A drop consisting of 2 μl protein solution was mixed with 2 μl precipitation solution and equilibrated against a 1 ml reservoir solution. The PPARγ LBD crystals with typical dimensions of 0.1 × 0.1 × 0.15 mm appeared in one week. Crystals of PPARγ-pioglitazone were grown in 0.1 M HEPES–NaOH pH 7.5, 17.5% PEG 8000, 10% EG. Crystals of PPARγ-lobeglitazone were grown in 0.1 M HEPES–NaOH pH 7.5, 17.5% PEG 8000, 10% DMSO. The crystals of the TZD-bound PPARγ LBD were cryoprotected in the reservoir solution supplemented with 10% glycerol and flash-cooled by immersion in liquid nitrogen. Crystals were preserved in a cryogenic N_2_-gas stream (~100 K) during diffraction experiments.

Diffraction data for PPARγ-pioglitazone and PPARγ-lobeglitazone crystals were collected at a wavelength of 0.98 Å using an ADSC Q315r CCD detector on the 5C beamline at Pohang Light Source (PLS), Pohang Accelerator Laboratory. All data were processed and scaled using HKL-2000 (HKL Research Inc.) and handled with the CCP4 program suite^39^. The structures of the pioglitazone- and lobeglitazone-bound PPARγ were determined by molecular replacement using the structure of the PPARγ LBD (PDB code: 4EMA) excluding the bound-rosiglitazone as a search model. The *F*_o_-*F*_c_ maps showed clear electron densities of PPARγ and the bound ligands. The nine residues at 264–272 (269–274 in B chain) in the Ω-loop and a few residues in the C-termini were not visible due to disorder and were not modeled.

The final model of PPARγ-lobeglitazone including two molecules of PPARγ LBD, two molecules of lobeglitazone, and water molecules was refined to R_work_/R_free_ values of 19.2%/22.3% at 1.7 Å resolution. Pioglitazone was visible only in the A chain of the PPARγ LBD homodimer. Electron densities of ligands in chain B were very weakly visible. Therefore, the ligand was not modeled in the B molecule of the PPARγ-pioglitazone complex. The structure of PPARγ LBD – pioglitazone was refined to R_work_/R_free_ values of 20.2%/23.2% at 1.8 Å resolution.

### Differential scanning fluorimetry

The stabilization of the PPARγ LBD by ligand binding was monitored by increase of melting temperatures using differential scanning fluorimetry (DSF)^25,40^. SYPRO orange is an environmentally sensitive dye. The unfolding process exposes the hydrophobic region of proteins and results in a large increase in fluorescence, which is used to monitor the protein-unfolding transition. DSF was performed using the CFX96 Real-Time PCR system (Bio-Rad) with a C1000 Thermal Cycler using FRET mode. The recombinant PPARγ LBD was pre-loaded with rosiglitazone, pioglitazone, or lobeglitazone during protein purification by applying the protein to size exclusion chromatography equilibrated with the buffer containing 60 μM of each TZDs. In a single well of a 96-well PCR plate, a 20 μL reaction solution contained 4 μM of PPARγ LBD, 6 μM of each TZD ligand, and 2 μL of 20 × SYPRO orange (diluted from 5000 × stock in DMSO). Owing to the high affinities of TZD ligands, the PPARγ LBD seemed to be almost fully occupied by each ligand, which allowed comparison of stabilization by different TZD drugs. Well plates were sealed with a transparent adhesive sheet. The real-time PCR machine was programmed to equilibrate samples at 25 °C for 5 minutes and then increase temperature to 80 °C at a rate of 1 °C/30 sec. The melting temperature of the protein was obtained as the lowest point of first derivative plot (d*F*/d*T*), as calculated by the software included with the RT-PCR system.

### Thermal denaturation tests of the PPARγ LBD

The apo or TZD-bound form of the PPARγ LBD was added to the SEC buffer containing 0 μM or 60 μM of each TZD ligand, respectively. In each PCR tube, 20 μl of reaction solution contained 0.1 mM of the purified PPARγ LBD and 60 μM of ligand. The samples were heat-treated using a PCR machine for 3 min at 15 temperature data points. The protein samples were then centrifuged at 13,000 rpm for 30 min to remove protein precipitate. The concentration of the soluble PPARγ LBD in the supernatant of each tube was measured by UV absorbance at the wavelength of 280 nm. The global curve fitting of the data points and the calculation of *T*_m_ values were done using software SigmaPlot.

### Computational docking for affinity estimation of TZDs

The structures of the PPARγ LBD complexed with lobeglitazone, pioglitazone and rosiglitazone (PDB id: 3EMA) excluding water molecules were used for calculations of the binding energies of the TZDs, using the software AutoDock Vina^28^. AutoDock Vina is implemented with an efficient scoring function for the estimation of protein-ligand affinity and a search algorithm for binding mode predictions. Since the high-resolution structures of the TZD-bound forms are available, the conformations of ligands and receptors were fixed and the program was run with “score only” option with an exhaustiveness value of 20. The dimensions of the search space centered in the ligand-binding pocket were 21 × 23 × 30 Å. The free binding energy values of the TZDs were converted to relative K_d_ values using the equation, ΔG = −*RT* ln *K*.

### Data availability

The coordinates and structure factors for the PPARγ LBD complexed with lobeglitazone and pioglitazone have been deposited in the RCSB Protein Data Bank with the accession codes, 5Y2T and 5Y2O, respectively.
